# Lysosome‐Targeted Bifunctional Therapeutics Induce Autodynamic Cancer Therapy

**DOI:** 10.1002/advs.202401424

**Published:** 2024-09-04

**Authors:** Athira Raveendran, Jinhui Ser, Seung Hun Park, Paul Jang, Hak Soo Choi, Hoonsung Cho

**Affiliations:** ^1^ Department of Materials Science and Engineering Chonnam National University Gwangju 61186 Republic of Korea; ^2^ Gordon Center for Medical Imaging Department of Radiology Massachusetts General Hospital and Harvard Medical School Boston MA 02114 USA

**Keywords:** anticancer agent, cell‐penetrating peptide, fluorescence dye, near‐infrared imaging, targeting therapy

## Abstract

Autodynamic cancer therapy possesses tremendous potential for enhancing therapeutic efficacy by initiating the treatment process autonomously within targeted cells. However, challenges related to biocompatibility and targeted delivery have hindered its clinical translation owing to the induction of adverse effects and cytotoxicity in healthy cells. In this study, a novel approach for auto‐initiated dynamic therapy by conjugating zwitterionic near‐infrared fluorophores to a cell‐penetrating peptide is proposed. This enables efficient cellular uptake and specific targeting of therapy to desired cells while avoiding off‐target uptake. The zwitterionic bioconjugate causes cancer‐specific toxicity following its internalization into the targeted cells, triggered by specific intracellular conditions in lysosomes. This innovative approach enables selective targeting of lysosomes in malignant cells while minimizing cytotoxic effects on normal cells. By targeting lysosomes, the method overcomes inherent risks and side effects associated with conventional cancer treatments, offering a selective and effective approach to cancer therapy.

## Graphical abstract

The study focuses on a novel auto‐initiated cancer therapy that employs zwitterionic near‐IR fluorophores attached to cell‐penetrating peptides for accurate targeting. This method guarantees efficient cellular absorption and precise targeting, leading to cancer‐specific toxicity within the lysosomes of malignant cells. This selective targeting improves the effectiveness of cancer treatments and reduces side effects, making it a highly desirable approach in oncology.

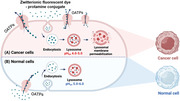

## 1 Introduction

Current cancer treatments, such as surgery, chemotherapy, radiotherapy, and phototherapy, have advanced rapidly following the efforts expended to combat the high mortality cancer rates.^[^ ^1^ ^]^ Despite prominent advances in targeted drug delivery and nanomedicine, cancer remains a challenging disease. Anticancer drugs or therapeutic approaches are being developed to enhance pharmacokinetics, including biodistribution and clearance, thereby increasing their efficacy in cancer treatment and their capacity to reduce systemic toxicity.^[^ ^2^, ^3^, ^4^, ^5^ ^]^ However, targeted drug delivery and therapy face limitations that reduce treatment efficiency. This necessitates the continuous pursuit of improved therapies and treatment strategies to enhance cancer management. Near‐infrared (NIR) fluorescent dyes have gained interest in recent years for cancer diagnosis and treatment owing to their potential as imaging and therapeutic agents.^[^ ^6^ ^]^ NIR fluorophores offer advantages, such as decreased tissue absorbance, scatter, and autofluorescence at NIR wavelengths, thus enabling deep tissue penetration with minimal background interference. Moreover, NIR dyes can be easily coupled with targeting moieties for specific targeting abilities.^[^ ^7^ ^]^ Nevertheless, when conjugated with chemotherapeutics and radiation treatments, NIR fluorophores can become lethal as they lack specificity for cancerous cells.^[^ ^8^, ^9^ ^]^

In this regard, a zwitterionic heptamethine indocyanine fluorophore (ZW800‐1C (ZW)), with its exceptional optical and physicochemical properties has exhibited tremendous potential as an excellent NIR fluorophore owing to its high‐molar extinction coefficient, quantum yield, high‐water solubility, and stability.^[^ ^10^, ^11^ ^]^ Moreover, owing to the balanced surface charges of ZW, background tissue uptake can be reduced, thus making it a popular choice for NIR fluorescence imaging.^[^ ^12^ ^]^ As it has no specific targetability, the tumor‐targeting ability of ZW is mainly dependent on the molecules attached to the fluorophore.^[^ ^13^, ^14^ ^]^ It is well known that zwitterionic or mixed charge conjugates are extremely sensitive to the acidic lysosomal pH environment, wherein cancer cells are more prone to aggregation and accumulation than normal cells.^[^ ^15^, ^16^, ^17^, ^18^ ^]^ Zwitterionic molecules, characterized by their equal but opposite charges, can adapt their configurations based on the pH levels, particularly becoming more protonated under acidic conditions. This protonation enhances their affinity for the negatively charged lysosomal membranes, promoting accumulation within these organelles. Once localized, the zwitterionic conjugate exploits the acidic conditions to initiate a therapeutic response targeted specifically at cancer cells, thereby minimizing the impact on healthy cells.^[^ ^18^ ^]^ Recent research supports this adaptive behavior of zwitterionic materials in biological systems, emphasizing their potential for targeted therapeutic applications.^[^ ^17^, ^18^ ^]^ These particles can rapidly aggregate and accumulate in the acidic, solid tumor lysosomes (pH_lys_: 3.4–4.7), compared with normal cell lysosomes (pH_lys_: 4.5–6.0),^[^ ^19^, ^20^, ^21^, ^22^ ^]^ thus causing LMP (lysosomal membrane permeabilization) and cell death.^[^ ^23^, ^24^, ^25^, ^26^ ^]^ Apoptosis and necroptosis (regulated forms of necrosis) are characterized by the disruption of lysosomal membranes by LMP and the consequent release of hydrolytic enzymes and proteins into the cytoplasm.^[^ ^26^, ^27^ ^]^ Consequently, direct or indirect lysosome‐targeting with various medications can be viewed as a highly target‐specific therapeutic approach for several diseases, including cancer.^[^ ^28^ ^]^

As cancer cells commonly overexpress cell‐surface receptors that bind peptides, these receptors are often used as targets for targeted tumor therapy.^[^ ^29^ ^]^ Protamine (Pro) is a positively charged cell‐penetrating peptide (CPP) composed of 4 arginine‐rich peptides with membrane‐translocating abilities, promoting endocytosis. Pro specifically accumulates in metabolically active lysosomes, and cancer‐associated lysosomes are known to be more acidic than their normal counterparts.^[^ ^28^, ^30^ ^]^

**Figure 1:**
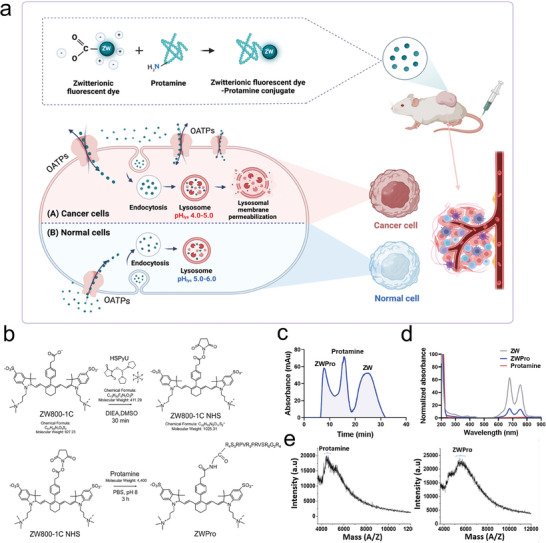
Mechanism of action and synthetic scheme of ZWPro. a) Schematic illustration of cellular uptake through organic anion transporting polypeptides (OATPs)‐mediated endocytosis of the anticancer ZWPro conjugates in cancer (A) and normal cells (B). b) Synthesis and characteristics of ZWPro. ZW800‐1C *N*‐Hydroxysuccinimide (NHS) ester reacts with the *N*‐terminal proline of protamine (Pro) to yield ZWPro. c) Fast protein liquid chromatography data shows 3 fraction peaks equivalent to ZWPro, Pro, and ZW. d) Optical spectra of ZW, ZWPro, and Pro. All optical properties were quantified using a 1X phosphate‐buffered saline (PBS) buffer solution. e) Matrix‐assisted laser desorption ionization‐time‐of‐flight‐mass spectrometry data showing Pro, ZW, and ZWPro. The shifted peak of ZWPro indicates successful conjugation.

In this study, we introduce a novel CPP (Pro)‐conjugated zwitterionic fluorophore (ZW), heron referred to as ZWPro, capable of specific lysosomal targeting and therapy. Upon accumulation in lysosomes, ZWPro disrupts the lysosomal membrane, causes the release of proteases, and induces lysosome‐dependent cell death in cancerous cells (**Figure** **1**a). This conjugate exhibits remarkable potential as a multifunctional agent, thus offering simultaneous tumor‐targeting, imaging, and anti‐cancer therapy options. This bifunctional anti‐cancer drug is anticipated to have profound implications in the field of oncology and is expected to open new avenues for effective cancer treatment.

## 2 Results

### 2.1 Synthesis and Characterization of ZWPro

The zwitterionic fluorophore ZW (emitting NIR fluorescence at 800 nm) was synthesized as reported previously.^[^ ^6^ ^]^ ZW demonstrated low‐plasma protein binding and a well‐balanced surface charge, thus leading to exceptional in vivo performance characterized by high selectivity for target tissues, improved bioavailability, and rapid renal clearance after conjugating with targeted ligands.^[^ ^13^, ^31^ ^]^ To create the ZWPro conjugate, ZW (1.2 equiv.) was covalently attached to Pro using *N*‐Hydroxysuccinimide (NHS) ester and led to a stable amide bond formation (Figure 1b). Pro peptides were entirely modified through a solution‐phase reaction at the *N*‐terminal of Pro. The ZWPro conjugate was subsequently purified using a fast protein liquid chromatography (FPLC) system to eliminate any unreacted ZW or Pro and byproducts. Figure 1c displays the separation of conjugate products by FPLC with distinct peaks corresponding to each fraction. ZWPro has the highest molecular mass and was eluted first, followed by Pro and ZW. The retention time profiles by high‐performance liquid chromatography (HPLC) have also confirmed it (Figure S1, Supporting Information). The absorbance peaks of ZWPro are the combinations of those for pure Pro and pure ZW, thus indicating that ZWPro possesses the characteristics of both ZW and Pro (Figure 1d). Using matrix‐assisted laser desorption ionization‐time‐of‐flight‐mass spectrometry (MALDI‐TOF‐MS) (Figure 1e) which confirmed the masses of Pro (molecular weight (MW) ≈4400 Da) and ZWPro conjugate (MW ≈5425.3 Da). And further confirmations were obtained 1H NMR data (Figure S2, Supporting Information) validated the successful conjugation process.

### 2.2 Optical Properties

The absorption and emission profiles of ZWPro, and the corresponding optical properties of Pro, ZW800‐1C, and ZWPro are shown in Figure 1d and Figure S3a (Supporting Information). For the in vitro stability test of ZWPro, we incubated ZWPro solution which dissolved PBS and 10% fetal bovine serum (FBS) in PBS at 37 °C, and measured absorbance spectra from 280–850 nm at each time point until 24 h (Figure S3b, Supporting Information). Fluorescence images and values are used to determine quenching concentration under a 760 nm channel of different concentrations from 0.8 to 50 µM of ZWPro between 0 to 4 h (Figure S3c, Supporting Information). The results show that ZWPro is stable and maintains its fluorescence at higher concentrations and does show some quenching at lower concentrations. We also conducted the Photostability test of ZW800‐1C and ZWPro in PBS (Figure S3d, Supporting Information) and confirmed the photostability of ZWPro in different solvents including Distilled water and 10% FBS in PBS (Figure S3e, Supporting Information). ZW showed strong photostability, retaining the intensity of its fluorescence in all tested solvents. When dissolved in phosphate‐buffered saline (PBS) and 10% FBS, ZWPro retained ≈70% of its initial fluorescence at the end of the testing period. Interestingly, in distilled water, ZWPro showed increased stability, retaining ≈90% of its fluorescence. These findings indicate that ZWPro has sufficient optophysical stability for potential biological applications.

### 2.3 In Vitro Cytotoxicity of ZWPro and ZW

**Figure 2:**
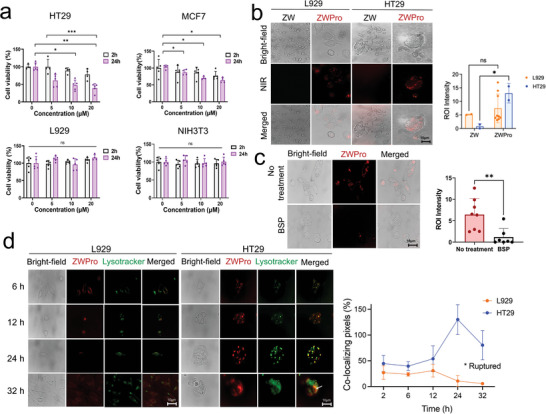
In vitro cellular evaluations of ZWPro in cancer cells. a) Cytotoxicity assay showing HT29, MCF7, L929, and NIH3T3 cells treated with 20 µM of ZW and ZWPro for 2 and 24 h, respectively, followed by an assessment of cell viability using the water‐soluble tetrazolium assay (*n* = 5, mean ± s.e.m.). b) Cellular uptake and intracellular endosomal localization of ZW and ZWPro were observed by treating HT29 and L929 cells with 20 µM of ZW and ZWPro. ZWPro was observed in the cells, while ZW was not internalized. The internalization index was determined in 2–9 photographic areas (ns, not significant, *p <  0.05 based on two‐way analysis of variance followed by Bonferroni's multiple comparison test). c) Inhibition assay of cellular uptake of ZWPro to determine the entry mechanisms in HT29 cells. Cultured cells were incubated with bromsulphthalein for 20 min and then incubated with ZWPro (20 µM) in media for 15 min. Data are presented as means ± standard deviations (*n* = 7–8) **p < 0.01 based on Welch's t‐test. d) Findings from HT29 human colorectal adenocarcinoma cells and L929 fibroblast cell lines co‐stained with the lysosome marker LysoTracker Green. Co‐localization occurs at specific time intervals during imaging. The co‐localizing pixels (%) were calculated by dividing the area of overlap between ZWPro and LysoTracker by the total area of the LysoTracker, respectively. Scale bars: 10 µm.

In Vitro, cytotoxic effects of ZW and ZWPro were assessed in both cancer and normal cells using the water‐soluble tetrazolium (WST)−8 assays (**Figure** **2**a). Cell viability was measured at 2 and 24 h after treatment with different concentrations of ZWPro (0, 5, 10, and 20 µM). As depicted in Figure 2a, treatment with ZWPro results in significant HT29 and MCF7 cancer cell death, while demonstrating minimal cytotoxicity toward L929 and NIH 3T3 fibroblast cells. Notably, ZWPro exhibited higher cancer‐killing efficacy in HT29 cells compared with MCF7 cells with cell death exceeding 60% at the 20 µM concentration in HT29 cells. Remarkably, even the 5 µM concentration of ZWPro induced efficient cell death in these cancer cell lines. Notably, 2 h after the onset of ZWPro treatment (5 µM) to HT29 and MCF7 cells, only a slight reduction in cell viability was observed in both cell cases, and no harmful effects were observed in noncancerous cells. Extended ZWPro treatment for 24 h at higher concentration (20 µM) demonstrated its remarkable selectivity in inducing targeted‐specific cytotoxicity on cancer cells (HT29: ***p < 0.001, MCF7: *p < 0.05). In contrast, ZW and protamine alone exhibited negligible toxicity across all treatment groups (Figure S4a,b, Supporting Information).

### 2.4 Cell Binding and Intracellular Localization

We also confirmed the specificity of the ZWPro conjugate for tumor targeting using the 2 cancerous cell lines, namely HT29 colorectal adenocarcinoma and MCF7 breast cancer cells. Two normal cell lines, namely L929 mouse fibroblast and NIH 3T3 mouse embryonic fibroblast cells, were used as controls. ZWPro treatment of cancer cells and normal cells demonstrated high interaction with biological membranes that facilitated extensive cellular uptake. In contrast, we detected almost no measurable fluorescence signal in unconjugated ZW‐treated groups (Figure 2b; Figure S5a, Supporting Information). To determine whether the specific uptake and accumulation of ZWPro in cancer cells were mediated by organic anion‐transporting polypeptides (OATPs), an inhibitor of OATP was pretreated with HT29 cells. Bromosulfophthalein (BSP), a competitive inhibitor of OATPs attenuated the uptake and retention of ZWPro by HT29 cancer cells (Figure 2c).

To determine the sites of ZWPro localization, cells were co‐stained with the lysosomal targeting dye LysoTracker to verify the lysosomal localization of ZWPro. ZWPro clearly shows colocalization with the lysosomal marker LysoTracker Green in merged images, thus implying that ZWPro has been internalized via the endocytic pathway and OATPs have selectively accumulated in lysosomes in both cancerous and normal cells (Figure 2d; Figure S5b, Supporting Information). To provide further insights into the specific internalization process and cancer‐selective cytotoxicity, colocalization imaging experiments with Lysotracker Green were conducted using fluorescence microscopy at specific time intervals. The total amount of internalized ZWPro conjugate seemed similar in cancerous and normal cells in the initial few hours after treatment. Interestingly, the fluorescent intensity in normal cells decreased 4 h post‐treatment of ZWPro, and ZWPro was completely removed from normal L929 cells 24 h post‐treatment. However, in HT29 cancer cells, ZWPro began to accumulate rapidly and appeared to increase considerably following the 24 h treatment time. The fluorescence intensity of ZWPro and Lysotracker Green diminished and diffused ≈32 h after the onset of treatment. This suggests that the cytotoxicity of ZWPro against cancer cells may originate from the lysosomal rupture that follows the lysosomal swelling and release of lysosomal proteases into the cytosol. Overall, the co‐localization imaging studies showed that the accumulation of ZWPro in the lysosomes of cancerous cells for a prolonged time and the clearance of the conjugate from normal cells are the major factors in the selective cytotoxicity of ZWPro against cancer cells (Figure 2a).

### 2.5 Flow Cytometric Analysis

**Figure 3:**
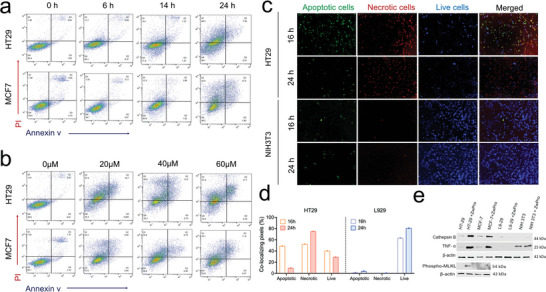
Annexin V/Propidium iodide (PI) double staining flow cytometric analysis at a) different time intervals or b) different concentrations of ZWPro after ZWPro treatment of HT29 and MCF7 cells for 24 h. c) Apoptosis/necrosis/live cell assay and fluorescence microscopy. HT29 and NIH3T3 cells were treated with 20 µM of ZWPro for 16 and 24 h. Images were acquired after co‐staining with apopxin (apoptosis detection dye, green fluorescence), 7‐AAD (necrosis detecting cell impermeable dye, red fluorescence), and CytoCalcein (live cell detection, blue fluorescence). d) Relative fluorescence intensity was calculated by dividing the area of apoptotic, necrotic, and live cells by the total number of cells, respectively. e) Western blot data show the expression of cathepsin B, TNF‐α, phosphorylated MLKL antibody, and β‐actin as the loading control. The samples were loaded as follows: HT29, HT29 + ZWPro, MCF7, MCF7 + ZWPro, L929, L929 + ZWPro, NIH3T3, and NIH3T3 + ZWPro.

To validate the cell death of cancerous cells upon treatment with the ZWPro conjugate, we used the annexin V/Propidium iodide (PI) staining method; fluorescence was assessed by flow cytometry. The control group (cells without treatment) displayed no background staining with either annexin V or PI. HT29 and MCF7 cells treated with 20 µM of ZWPro for 6, 14, 24, and 32 h showed a gradual increase in the number of PI‐positive cells over time (**Figure** **3**a). After 32 h of treatment, almost 80% of HT29 cells and 70% of MCF7 cells were stained with PI, which indicated necrotic cell death. Additional analysis with higher concentrations of the conjugate (20, 40, and 60 µM) after 24 h of treatment showed nearly identical amounts of the PI‐stained cell populations (Figure 3b); this finding indicates that ZWPro cytotoxicity in the cancer cell was primarily dependent on the treatment time rather than on the increased concentrations.

### 2.6 Cell‐Death Mechanism

Our results demonstrated that ZWPro exhibited cancer‐specific cytotoxicity mediated by its specific pattern of accumulation in cancer and normal cells. The prolonged accumulation of ZWPro may be attributed to the acidic pH of cancerous cells, and the accumulation or aggregation of these mixed charge particles may cause the destabilization and functional impairment of the cancer lysosomes. In contrast, owing to the higher pH environments of the lysosomes of normal cells, ZWPro remains a small particle and is excreted within a few hours following internalization, thus resulting in selective cancer toxicity. Our co‐localization studies using the LysoTracker Green and ZWPro show the swelling of lysosomes followed by the diffusion of fluorescence; lysosomal swelling may lead to cell death attributed to LMP (Figure 2d). The presence of LMP was demonstrated by western blotting, which showed an increased cathepsin B expression after 24 h of the ZWPro treatment (Figure 3e). To elucidate the ZWPro‐induced cell death signaling pathway, HT29, MCF7, L929, and NIH3T3 cells were treated for 24 h with ZWPro, and the expression of tumor necrosis factor (TNF‐α) and phosphorylated mixed lineage cathepsin B were examined by western blotting. Increased expressions of TNF‐α and cathepsin B were found in HT29 and MCF7 cells after ZWPro treatment compared with the control group. In contrast, the expressions of TNF‐α and cathepsin B in normal cells were almost similar compared with those of the treatment and control groups. This indicates lysosomal rupture and protease release (mainly cathepsin B) leads to lysosomal‐dependent cell death. There is abundant evidence that suggests that lysosomal membrane damage, such as damage by LMP, can trigger the release of hydrolytic enzymes, particularly cathepsin B, which in turn trigger necroptosis.^[^ ^12^ ^]^ The activation of phosphorelated (P)‐MLKL in HT29 and MCF7 cancer cells leads to TNF‐α–induced, receptor‐interacting protein kinase 1 (RIPK1)/RIPK3/MLKL‐dependent necroptotic cell death. The binding of TNF‐α to TNF receptor 1 (TNFR1) leads to the activation of a cell death signaling pathway,^[^ ^13^ ^]^ which induces interactions with several proteins to form a complex.^[^ ^14^ ^]^ Imaging (Apopxin Green Indicator for apoptotic cell staining and 7‐AAD (7‐Aminoactinomycin D) for necrotic cell staining) using fluorescent microscopy showed both apoptotic and necrotic dye staining patterns following 16‐h treatments. Most cells in the experiment were only stained by 7‐AAD after 24 h of treatment (Figure 3c). According to the co‐localizing pixel results, 16 h treatment of the HT29 group with ZWPro shows both apoptotic and necrotic signals compared with the 24‐h treatment group and other L929 treatment group findings (Figure 3d). After 16 h treatments of the HT29 cells with ZWPro, the proportion of necrotic cells was higher than that of apoptotic cells. Apoptosis/necrosis imaging detected cells demonstrating both the features of apoptosis and necrosis suggesting necroptotic cell death.

### 2.7 In Vivo Tumor Reduction Study

**Figure 4:**
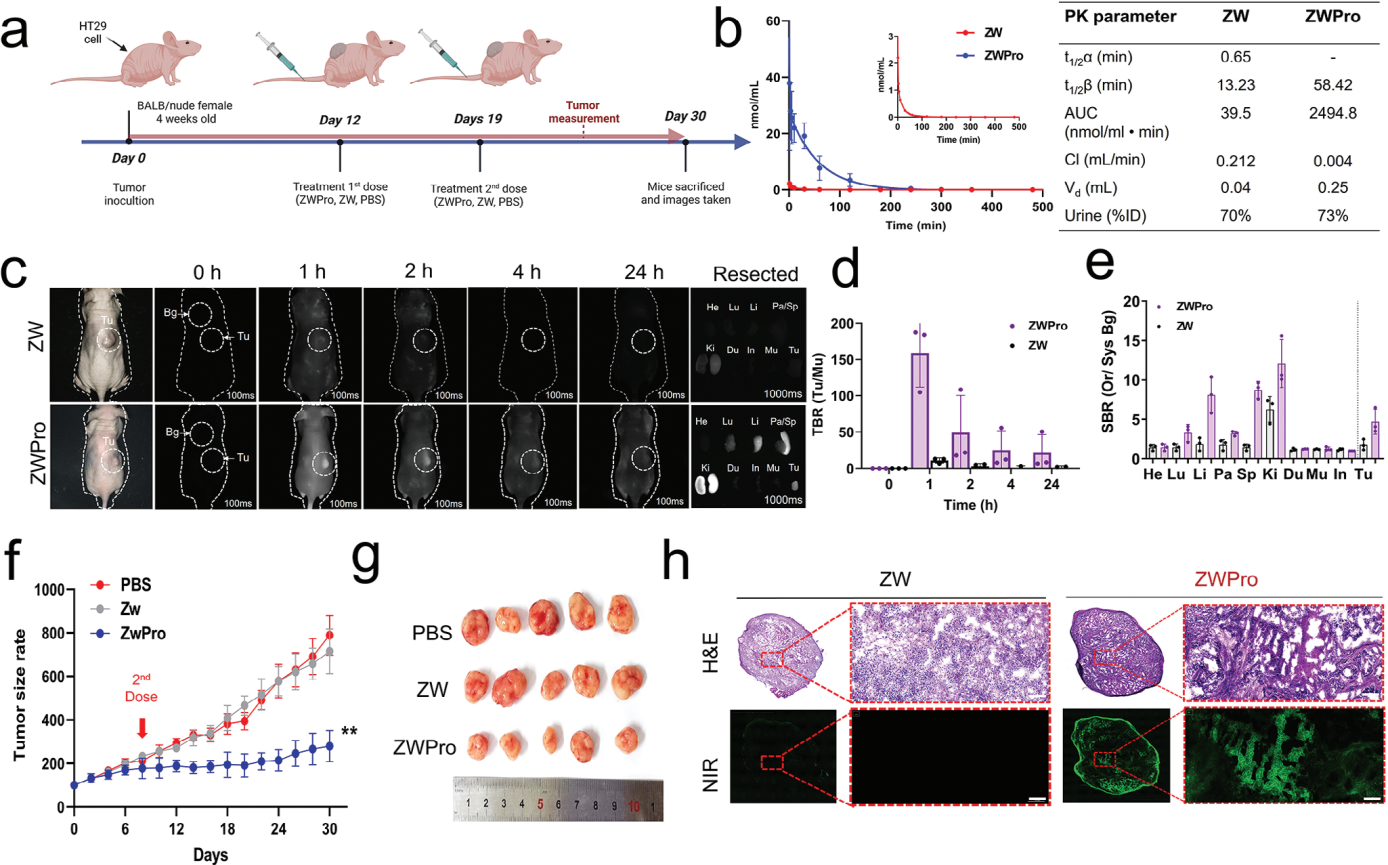
In vivo, tumor growth inhibition and studies in tumor‐induced mice subcutaneously injected with HT29 cells and treated with ZWPro. a) Schematic of the treatment schedule and experimental design of PBS, ZW, and ZWPro. Biodistribution and pharmacokinetic (PK) parameters of targeted ZW and ZWPro in normal mice. PK parameters of ZW and ZWPro (0.3 µmol kg^−1^) (t1/2α; distribution half‐life, t1/2β; elimination half‐life, AUC; area under the curve, Vd volume of distribution, and urinary excretion (% injected dose, %ID)) were calculated using the software Prism 9. Urinary excretion occurred at 48 h post‐injection (*n* = 2–3 per group, mean ± s.e.m.). (b) In total, 20 nmol of each compound was injected into BLAB/C nude mice (weights = 20–25 g) and time‐dependent imaging was performed post‐injection. c) Color and NIR fluorescence in vivo images of ZW and ZWPro (0.3 µmol kg^−1^) were taken at different time intervals. d) Tumor‐to‐background (TBR) ratios of tumors (white dotted circles in (c)) compared with muscles obtained from at each time point; and e) signal‐to‐background (SBR) outcomes were calculated using NIR images of resected organs at 48 h post‐injection. (Exposure time = 100 ms, *n* = 3, mean ± s.e.m.) The SBR was calculated by the fluorescence intensity of each organ (Or) against the system background (Exposure time = 1000 ms, *n* = 3, mean ± s.e.m.). Scale bar: 1 cm. f) Tumor reduction assay of the PBS, ZW, and ZWPro. g) Photographs of the dissected tumor tissues. h) Postoperative histopathological examination; H&E stained images and NIR fluorescence microscopy images. Abbreviations used are: He, heart; Lu, lungs; Li, liver; Pa, pancreas; Sp, spleen; Ki, kidneys; In, intestine; Mu, muscle; Tu, tumor. Data are presented as mean ± standard deviation (SD) (Scale bar: 100 µm, *n *= 5, ***p < 0.001, **p < 0.01).

Owing to the cancer‐specific cytotoxicity of ZWPro, the in vivo tumor targetability and theranostic capacity of ZWPro were evaluated in HT29 tumor‐bearing mice. Preliminary studies were conducted to determine the optimal dosage of ZWPro to achieve effective anticancer activity. ZWPro was found to reduce tumor size effectively (dose of 50 nmol at 9.5 mg k^−1^g), with 2 doses administered on the first day of treatment and 7th day of treatment. To investigate the antitumor impact, mice were intravenously injected with PBS (150 µL) (control group), and with ZWPro (150 µL) or ZW (150 µL, 50 nmol at 9.5 mg k^−1^g). The intravenous injection was administered twice to a total dose of 19 mg k^−1^g, as shown in **Figure** **4**a. ZWPro significantly reduced tumor growth in mice compared with the PBS and ZW groups (Figure 4f,g). The tumor size increased slightly after the treatment on d 0. However, it was significantly inhibited after the d 7 treatment, thus demonstrating the outstanding antitumor activity of ZWPro. To ensure that ZWPro did not promote weight changes, mice were weighed every other day for 30 days. In comparison to the control group, neither ZWPro nor ZW resulted in weight loss (Figure S6, Supporting Information). Stable mouse body weights were observed across all experimental groups. After 30 d of treatment with ZWPro, tumors were smaller than those in the ZW and PBS groups (Figure 4g).

### 2.8 Time‐Dependent In Vivo Tumor Imaging and Biodistribution of ZWPro

The in vivo tumor‐targeting ability of ZWPro was evaluated by injecting 20 nmol of ZWPro and ZW intravenously into HT29 tumor‐bearing mice and then monitoring them with a real‐time NIR fluorescence imaging system at regular time intervals for 24 h (Figure 4c). Time‐lapse NIR fluorescence imaging of ZWPro‐injected mice showed fast tumor accumulation 2 h after injection with no nonspecific uptake. ZW alone showed no tumor specificity or cell penetration ability, which is consistent with its well‐known properties reported previously.^[^ ^15^ ^]^ High‐fluorescence intensity was maintained at the tumor site after ZWPro injection for ≈24 h followed by a minor decrease. The prolonged accumulation of ZWPro at the tumor site may contribute to its cancer‐specific cytotoxicity and can be used for successful real‐time tumor imaging. The biodistribution of ZWPro was studied by evaluating the differences in fluorescence signals between various organs 6 h after injection. The pharmacokinetic parameters of ZWPro were further evaluated after a single intravenous injection. The blood concentration decay curve yielded a blood half‐life of 58.42 min and an area under the curve of 2494.8 nmol mL^−1^ min^−1^ (Figure 4b), thus indicating that ZWPro was rapidly excreted through bile and urine. A longer blood half‐life allows the ZWPro to remain in circulation for a longer duration. Continuous exposure to the ZWPro at effective concentrations can enhance its overall efficacy in inhibiting tumor growth or inducing tumor cell death. This prolonged exposure can lead to sustained suppression of tumor cell proliferation or other desired therapeutic effects. Interestingly, ZWPro did not exhibit any nonspecific tissue or organ uptake after 48 h following injection owing to its fast renal elimination, which is very similar to the superior in vivo performance of ZW800‐1, as previously described.^[^ ^8^ ^]^ ZWPro possesses a net positive surface charge, unlike the balanced net charge of ZW, which may contribute to the high‐tumor targetability of ZWPro. The *ex vivo* imaging results also showed an excellent accumulation of ZWPro in the tumor (Figure 4c; Figure S7a,b, Supporting Information). The quantification of fluorescence signals in major organs showed that the ZWPro uptake in tumor tissues was much higher than that in other vital organs. Results from biodistribution studies have confirmed that ZWPro can selectively target tumors and localized tumor tissues while being eliminated from normal organs. The tumor‐to‐background (TBR) and signal‐to‐background (SBR) responses demonstrated a higher accumulation of ZWPro in the cancer cells with minimum background signals. Subsequently, we evaluated the tumor targetability of ZW and ZWPro in a xenograft colon cancer animal model. High‐magnification images show accumulation of ZWPro in the cytoplasm of the tumor cells and relatively homogeneous distribution throughout the tumor tissue, thus indicating that this compound can penetrate deep into tumor tissue via diffusion mechanisms and enter cancer cells. We evaluated the penetration and distribution of ZW and ZWPro by fluorescence microscopy and confirmed tumor, kidney, and liver morphological changes using hematoxylin and eosin staining (H&E) (Figure 4h; Figure S8, Supporting Information). The entire tumor tissue image of the ZW treatment group doesn't show any significant fluorescence signals. In contrast, the tumor tissue image of the ZWPro treatment group shows that there are apoptotic cells with condensed cytoplasm, condensed and hyperchromatic chromatin, and fragmented nuclei within the interstitial space. Even ZWPro accumulates kidney and liver, it does not affect normal cells and organs, only toxic to tumor tissue in mice (Figure S8, Supporting Information). Moreover, necrosis was identified in the center of the tumor which led to many areas with empty/low cell populations. Additionally, according to NIR microscopic findings, substantial ZWPro fluorescence signals were observed in the deep and boundary tumor tissues, and across the entire tumor specimen. Previous research suggested that an amine group alteration in the structure of ZW800‐1 can play a critical role in generating tumor‐targeting ability.^[^ ^9^ ^]^ Considering this, we suggested that the amine group attached to the ZW structure after the conjugation with Pro resulted in the net positive charge, and played a substantial role in the efficient tumor targeting of ZWPro.

## 3 Discussion

In this study, we designed and developed a novel approach for cancer‐lysosome‐targeted therapy that used a zwitterionic fluorescent dye and cell‐penetrating peptide conjugate that has cancer‐selective accumulation and cytotoxicity properties. According to our understanding, this represents the first research study on the zwitterionic NIR fluorophore (ZW800‐1C) and a cell‐penetrating peptide created as an anticancer agent that can be employed in cancer therapy without the need for light or radiation for activation and efficient cell death. Hence, when the neutral‐charged ZW (+3/−3) conjugates with the positively charged Pro through a stable amide bond, the neutral charge distribution of ZW is disturbed by the dominance of the positive charge.^[^ ^9^ ^]^ Owing to this property of zwitterionic particles, we anticipated that the high specificity of the developed ZW conjugate with Pro toward tumors would result from pH‐dependent accumulation events. More importantly, the synthesized compound not only accumulated in tumors without nonspecific uptake but also produced an anticancer effect by selectively destroying the tumor.

While the ZWPro conjugate shows promising specificity and cancer‐selective accumulation via OATPs and showed anticancer efficiency by pH‐dependent mechanisms, its targeting efficiency may be further improved by activatable targeting mechanisms. Activatable systems, such as those responsive to tumor‐specific enzymes or hypoxia, can provide additional specificity by ensuring that therapeutic effect is initiated only within the target tissue. For instance, enzyme‐responsive peptides that are cleaved by matrix metalloproteinases (MMPs) overexpressed in the tumor microenvironment have shown enhanced targeting capabilities.^[^ ^32^ ^]^ Similarly, pH‐sensitive systems that exploit the acidic nature of the tumor microenvironment can improve the selective release and activation of therapeutic agents, particularly inside the target tissue.^[^ ^33^ ^]^ Such strategies could complement our lysosome targeting approach enhance the efficiency and minimize off‐target effects.

The in vitro studies showed that ZWPro was internalized via endocytosis in both normal and cancer cells, followed by its accumulation in lysosomes. ZWPro also showed endogenous affinity to OATPs, which were overexpressed in cancer cells, and entered cancer cells primarily via OATPs; it was retained in the lysosomes for a long time. A remarkable cascade of targeted delivery and a distinct prolonged accumulation pattern of the conjugate in cancer cells resulted in cancer‐specific toxicity. Furthermore, we observed that increased accumulation of the ZWPro conjugate, particularly in cancer cells for a prolonged time, caused lysosomal swelling, a gradual reduction of lysosomal membrane integrity, rupture, and the release of lysosomal proteases to the cytosol, which triggered cellular responses and led to necroptotic cell death. In parallel, ZWPro aggregation in lysosomes appeared restricted in normal cells and ZWPro was expelled from the lysosome after a few hours of treatment without toxicity effects. Further, the flow cytometric data for cell death (Annexin V/PI) showed prominent PI staining patterns in ZWPro‐treated cells indicative of apoptotic cell death. The cancer‐selective cytotoxicity resulted from the prolonged accumulation pattern of the conjugate in cancerous cells; by contrast, the conjugate was rapidly excluded from normal cells. However, the fluorescence microscopy investigation of apoptotic and necrotic cell death (Apopxin/7‐AAD/CytoCalcein assay) revealed both apoptotic and necrotic cells following a 16 h treatment with ZWPro in cancer cells. In contrast, after the 24 h treatment, most cells seemed to die owing to apoptosis and necroptosis (Figure 3c). Studies showed that phosphatidylserine, which binds to the apoptotic staining dye, translocates to the outer plasma membrane before membrane integrity is compromised. This could explain why we found apoptotic and necrotic cells following the 16‐h ZWPro treatment. The binding of the apoptosis‐staining dye to phosphatidylserine at the cell surfaces of apoptotic cells is the mechanism based on which apoptosis is detected. Additionally, cell‐impermeable dyes, like PI, are used to detect necrotic cells by revealing enhanced permeability patterns. Both of these characteristics can be found in apoptotic cell death. We confirmed the possibility of a ZWPro‐induced cancer cell death pathway by evaluating the expression of different proteins involved using western blotting. Moreover, we observed an increased expression of TNFα in ZWPro‐treated cancer cells. Activation of apoptosis by TNFα is well established. TNF‐induced apoptosis is known to occur through a well‐known molecular mechanism by the cooperation of TNFα and cathepsin B to coordinate numerous downstream signaling pathways.^[^ ^34^, ^35^ ^]^ Necroptosis is shown to be mainly activated by P‐MLKL.^[^ ^36^, ^37^ ^]^ Based on these findings, we conclude that ZWPro triggered necroptosis via a TNF‐mediated mechanism.

While our research shows that the zwitterionic bioconjugate is effective in preclinical cell models, additional testing in complex biological systems is necessary to determine its potential for translation. Future studies should focus on evaluating the therapeutic effects of the bioconjugate in complex animal models to assess its pharmacokinetics, biodistribution, and in vivo toxicity. Additionally, examining the efficacy of the bioconjugate in primary human samples will provide valuable insights into its clinical relevance. These steps are necessary to pave the way for potential clinical trials and therapeutic applications. In conclusion, we developed a multifunctional, lysosome‐targeted, anticancer therapeutic agent for tumor imaging with tumor‐targeting capabilities that effectively destroyed cancer cells. The conjugate of the zwitterionic fluorophore and cell‐penetrating peptide with mixed charge characteristics showed a cancer‐specific accumulation in the lysosome, which ultimately led to cell death by LMP. Thus, this drug‐free, lysosome‐targeted, light‐independent self‐triggered autodynamic therapy is capable of diminishing the negative effects of light irradiation treatments and off‐target toxicity caused by chemotherapeutic drugs. This study demonstrates a practical method for the development of smart anticancer agents with the potential to target and eradicate cancer cells selectively while causing minimal harm to normal cells.

## 4 Experimental Section

ZW800‐1C was provided to us by Professor Hak Soo Choi. Protamine sulfate (Hanlim Pharmaceutical Co., Ltd., Seoul, Republic of Korea; No. 339, MW = 4.4 kDa), Dimethyl sulfoxide anhydrous (DMSO; Life Technologies, Inc., Carlsbad, CA, United States of America (USA); No. LOT. 0305C186), AKTA Prime plus Fast Protein Liquid Chromatography (FPLC; General Electric (GE) Healthcare Lifesciences, Marlborough, MA, USA), and PBS (10X, pH 8 (ThermoFisher Scientific, Waltham, MA, USA; No. LB 204‐02) was purchased from various manufacturers.

### Cell Culture and Biological Experiments

HT29 (colorectal adenocarcinoma cells), MCF7 (breast cancer cells), L929 (mouse fibroblast cells), and NIH3T3 (mouse embryonic fibroblasts) were obtained from the American Type Culture Collection (ATCC, Manassas, VA, USA). McCoy's 5A Medium Modified, Roswell Park Memorial Institute 1640 medium, fetal bovine serum (FBS), penicillin–streptomycin, trypsin‐ ethylenediaminetetraacetic acid purchased from Gibco (Thermo Fisher Scientific), Lysotracker Green (ThermoFisher Scientific; No. L7526), and EZ‐Cytox (WST; Seoul, DogenBio, Republic of Korea; No. EZ‐1000), were used in this research. Clarity Western enhanced chemiluminescence (ECL) substrate (BIO‐RAD, Republic of Korea, No. 1 705 061), radioimmunoprecipitation assay buffer (RIPA) lysis buffer (ChemCruz, USA; No. SC‐24948A) Cathepsin B recombinant rabbit monoclonal antibody (ThermoFisher Scientific; No. WA3178253), Phospho‐MLKL Rabbit mAb (Cell Signaling Technology, MA, USA), anti‐TNF alpha antibody (Abcam, Waltham, MA, USA; No. ab183218), anti‐beta actin antibody (Abcam; No. ab8227), goat anti‐rabbit IgG (Abcam; No. ab205718), SeeBlue Prestained standard (1X) (ThermoFisher Scientific; No. 2 534 775), Apoptosis/Necrosis Assay Kit (Abcam; No. ab176749), normal horse serum for blocking (Abcam; No. ab7484), PI (ThermoFisher Scientific; No. P1304MP), and Annexin V conjugates (ThermoFisher Scientific; No. A13201) were also employed for various assays.

### Synthesis and Characterization of ZWPro

Protamine sulfate was diluted (Pro; 14 mg, 3.415 µmol) in 1X PBS in a round bottom flask. A magnetic stirrer was used to adjust pH in the range of 8.0–9.0. ZW800‐1C NHS ester (ZW; 10.5 mg, 10.245 µmol in DMSO) was added dropwise into the Pro to attain a ZW:Pro ratio of 3:1 with vigorous stirring. The reaction occurred in a vial at 27 °C for 4 h using a digital rotator; the precipitate was obtained using the reaction mixture following the addition of excess acetone. The precipitates were centrifuged (3500 revolutions per minute (rpm) for 10 min) and the supernatant was discarded. The resulting pellet in dH_2_O was resuspended and reprecipitated with acetone. Steps 4 to 6 were repeated with acetone/EA, in sequential order. After the final washing with acetone, the residual solvents were dried in a vacuum. Two HiTrap desalting columns packed with GE Healthcare's Sephadex G‐25 superfine‐sized exclusion medium were used for the purification of the ZWPro conjugate using FPLC. The column was loaded with 1X PBS buffer solution before separation. Purification by AKTA Prime Plus was performed at room temperature with conductivity and ultraviolet light monitoring. Throughout the purification process, a gradient flow rate of 1 mL mi^−1^n was maintained, and 0.3 mL fractions were collected. Following purification, measurements using size exclusion chromatography (SEC) analysis on an Analytical HPLC system consisting of a 1260 binary HPLC pump with a 1260 ALS injector, a 35900E photodiode array detector (PDA; Agilent, 200–800 nm), and a 2475 multiwavelength fluorescence detector (Waters, Ex 770 nm and Em 790 nm) were performed. The column eluent was divided into 2 using a flow splitter (Upchurch Scientific, VWR, No. 53500–630). A part of the eluent flowed into the PDA equipped with an ultrahydrogel 2000 (7.8 × 300 mm) SEC. The mobile phase was maintained in PBS for 30 min and the flow rate was 0.75 mL mi^−1^n. Additionally, MALDI‐TOF‐MS analyses on all fractions to identify the conjugation of ZWPro were conducted.

The optical properties of ZW800‐1C, Pro, and ZWPro, their absorbance and fluorescence spectra, were performed across a wavelength range of 280 nm to 850 nm. Fluorescence spectra were acquired using an excitation set at 760 nm to align with the absorption peak of ZW800‐1C and ZWPro; emission was recorded from 280 nm to 850 nm to evaluate their emission properties. All optical properties measurements were used by BioTek Cytation 5 (Winooski, VT, USA) and UV‐Vis‐NIR spectrophotometer (USB‐ISS‐UV/VIS, Ocean Optics, Dunedin, FL, USA). To determine the quenching concentration, fluorescence images of 0.8 to 50 µM ZWPro solution in distilled water under a 760 nm laser with 0.4 mW cm^−2^ power were used.

Photostability assay was conducted to compare ZW800‐1C with ZWPro in PBS and also evaluated ZWPro in distilled water and 10% FBS in PBS exposed to continuous irradiation using a 760 nm laser diode at 4 mW cm^−2^, with white light (400–650 nm) at 40 000 lux and imaged up to 4 h. The fluorescence intensity (FI) signal (%) was calculated by measured values with ImageJ version 1.53t using images captured at each time point. Fluorescence values of the quenching assay were conducted at different concentration points at 0 and 4 h to determine the quenching concentration and to assess any significant photodegradation or photobleaching effects.

The data, presented as mean ± SD, assessed the fluorescence image by measuring regions of interest (ROI) and comparing fluorescence intensity against the initial fluorescence signal.

### Cellular Uptake of ZWPro and ZW

Fluorescence microscopic assessments were performed to compare the intracellular uptake of ZW and ZWPro in cancerous and normal cells. Two sets of cathepsin B‐expressing cancer cells (HT29 and MCF7) and 2 sets of normal cells (L929 and NIH3T3) were seeded in a confocal dish and incubated at 37 °C for 1 d before treatment. Subsequently, 20 µM ZWPro and ZW were added to the cells for 2 h. Subsequently, 50 nM of LysoTracker Green (a fluorescent dye that selectively labels lysosomes) was added for 30 min. Following incubation with the dye, the cells were resuspended with fresh media after they were rinsed 3 times with 1X PBS to remove any remaining dye, and imaging was performed. To determine the role of organic anion transporter peptides (OATPs) in the ZWPro uptake process, HT29 cells were pretreated with BSP (250 µM, Sigma–Aldrich, 167 207) for 20 min in the inhibition assay. Cells were then incubated with 20 µM of ZWPro at 37 °C for 15 min. After washing, cell images were obtained. Images were obtained using a Nikon Eclipse inverted microscope, DS‐Ri2, or DS‐Qi2 (Ex/Em, 754/774 nm; exposure time, 2 s). The fluorescent intensity of each cell was measured using ImageJ (version 1.51j8, National Institutes of Health, Bethesda, MD, USA). The outline of each cell was determined on a phase‐contrast image.

### In Vitro Cytotoxicity Assays

To evaluate the cytotoxicity of ZW and ZWPro in cancer and normal cells, HT29, MCF7, L929, and NIH 3T3 cells were collected by trypsinization and resuspended in 96‐well plates at a concentration of 10 000 cells/well in fresh growth media and incubated for 1 d at 37 °C under 5% CO_2_ conditions. After the cells were completely attached to the walls, the original medium was discarded and replaced with a 200 µL fresh culture medium which contained different concentrations of ZW and ZWPro. Cells were then cultured in a constant temperature incubator for 2 and 24 h. EZ‐Cytox solution (10%) was then added to each well, and the plates were incubated at 37 °C in a 5% CO_2_ incubator for 2 h in the dark. Cells were then exposed to a digital orbital shaker (Scilab Instruments Co, No. SSO‐2D) at 200 rpm. Absorption was subsequently measured at 450 nm on a microplate reader (Thermo Scientific, Varioskan LUX multimode). All data were demonstrated as means ± SD. Statistical analysis was conducted using the software GraphPad Prism 8.

### Protein Identification by Western Blotting

HT29, MCF7, L929, and NIH 3T3 cells (1 × 10^6^ cells) were treated with 20 µM of the ZW and ZWPro for 24 h. The cells were harvested and lysed in RIPA lysis buffer (500 µL). The cells were then centrifuged for 15 min at 10 000 g at 4 °C after incubation for 15 min on ice. Soluble proteins were then extracted from the supernatant and the Bradford assay was used to quantify the protein content. ZW and ZWPro treated with 20 µg of the cell protein lysates were loaded and separated by sodium dodecyl sulfate‐polyacrylamide gel electrophoresis along with the controls. The separated proteins were then transferred to polyvinylidene fluoride membranes. They were then blocked with 5% normal horse serum or 1% milk in autoclaved PBS 1X buffer for 1 h and then treated overnight at 4 °C with primary antibodies and gentle shaking. Excess antibodies were removed with tris‐buffered saline and Tween 20 (TBST). Subsequently, the membranes were incubated with the secondary antibody for a period of 2 h at room temperature (28 °C) and then washed multiple times with TBST to remove any remaining secondary antibodies. Signals from the bound antibodies were detected with the ECL kit.

### Apoptosis/Necrosis Assay

We used the apoptosis/necrosis assay kit for mammalian cells (HT29 and MCF7) for tests. The cells were seeded at a density of 1 × 10^6^ per well in 96‐well plates and incubated for 24 h at 37 °C under 5% CO_2_. Subsequently, 20 µM of the ZWPro was added and incubated for 16 h in 100 µL of the cell culture medium. After the treatment period, the medium that contained the ZWPro medium was removed, and the cells were washed with 100 µL assay buffer by carefully pipetting up and down. The cells were then treated with a mixture of Apopxin, a phosphatidylserine sensor (apoptotic cells, green fluorescence), the membrane‐impermeable dye 7‐AAD (necrotic cells, red fluorescence), and CytoCalcein Violet 450 (live cells, blue fluorescence). The cells were incubated at room temperature in dark conditions for 60 min. Cells were then carefully washed once with the 100 µL assay buffer and immersed in another 100 µL assay buffer. Fluorescent microscopy was used to evaluate the cells. Apoptotic cells yielded green staining outcomes owing to the binding of the Apopxin Green indicator to phosphatidylserine. They were visualized using the fluorescein channel (Ex/Em = 490/525 nm); necrotic cells were visualized using the red channel (Ex/Em = 550/650 nm), and live cells were visualized using the violet channel (Ex/Em = 405/450 nm). The fluorescent intensity of each cell was measured using ImageJ. The outline of each cell was determined using phase‐contrast images.

### Flow Cytometric Analysis

HT29 and MCF7 cells were seeded in 6‐well plates at a density of 1 × 10^6^ per well and incubated overnight. After incubation for 1 day, cells were treated with 20 µM of ZWPro for 6, 14, and 24 h. The cells were collected following trypsinization from each well and then centrifuged at 1000 rpm for 5 min. The cells were then centrifuged at 2000 rpm for 5 min at room temperature and washed twice with cold 1X PBS. Cells were then collected and washed with cold PBS at 4 °C and were then centrifuged at 1200 rpm for 3 min. The resulting pellets were then mixed with the 400 µL of the Annexin binding buffer (Invitrogen, No., V13246), PI (5 µL, Invitrogen, No., P1304MP), and Annexin V conjugates for apoptosis detection (Annexin V; 5 µL, Invitrogen, No, A13201) were added to the suspension and mixed gently, then incubated for 20 min at 4 °C in the dark. Cells were analyzed using flow cytometry (NaviosTM, Beckman‐Coulter, Miami, FL, USA). Finally, the cells and controls were analyzed in a flow cytometer, and the raw data were processed using the software FlowJo (version 10.0, manufacturer, city, state, country). Cells that were PI‐negative and Annexin V‐negative were considered to be live, PI‐negative and Annexin V‐positive cells were considered apoptotic, and cells that were positive for PI were considered necrotic.

### In Vivo Biodistribution and Pharmacokinetics of ZWPro

Animals were housed in an AAALAC‐certified facility and were studied in accordance with the institutional protocol (2016N000136) approved by the Massachusetts General Hospital Institutional Animal Care and Use Committee. Four‐week‐old NCr nu/nu mice (female; 17–20 g) were purchased from Taconic (Taconic Farms, Germantown, NY, USA). To establish tumor‐xenografted nude mice, HT29 cells were cultured in Mccoy's medium with 10% FBS and 1% penicillin and streptomycin. HT29 colon carcinoma tumors in mice were established by subcutaneously injecting 3 × 10^6^ resuspended in 100 µL of PBS/Matrigel (50 v/v%) at the flank. Before the onset of the experiment, tumors were allowed to reach an approximate volume (V) of 100 mm^3^. The length (L) and width (W) of the tumors were measured with a digital caliper and the size was calculated by the equation V = ½ LW^2^. In vivo imaging studies were conducted by administrating ZW and ZWPro intravenously at a concentration of 20 nmol in PBS (150 µL). Mice were anesthetized with Isoflurane levels of 1.5%–2% at a flow rate of 0.4–0.8 liter min^−1^ and Oxygen. Blood (less than 0.1 mL) was sampled in capillary tubes (Fisher Scientific, Pittsburgh, PA, USA) at the 0 min time point by slightly puncturing the end of the tail. Blood samples were obtained at 1, 3, 5, 10, 30, 60‐, 120‐, 180‐, and 240‐min post‐injection from the tail vein, and the fluorescence intensities of serum samples in capillary tubes were measured to calculate the distribution (t1/2α) and elimination (t1/2β) half‐life values (n = 2–3 for each group). The fluorescent signal of ZW and ZWPro in mice was observed with a real‐time NIR fluorescence imaging system (K‐FLARE) at different time points (2, 4, 6, 9, 24, and 48 h) after injection. After 48 h post‐injection, mice were sacrificed to harvest and image organs (heart, lung, liver, spleen, pancreas, kidney, intestine, skin, tumor). Results were presented as biexponential decay curves using the GraphPad Prism software (version 8.0, GraphPad, San Diego, CA, USA).

### In Vivo Tumor Reduction Test

The Chonnam National University Medical School Research Institutional Animal Care and Use Committee approved all the animal experiments. All experiments were conducted in accordance with the National Institutes of Health's guidance for the care and use of laboratory animals (CNU IACUC‐YB‐2022‐64). For the in vivo tumor targeting and tumor reduction studies, 4‐week‐old female BALB/c nude mice (17–20 g) were purchased from Orient Bio (Seongnam, South Korea). Mice were housed in a pathogen‐free environment with filtered top cages and provided sterile food and water. HT29 colon carcinoma tumors in mice were established by subcutaneously injecting 3 × 10^6^ resuspended in 100 µL of PBS at the hindlimb. Before the onset of experiments, tumors were allowed to reach a size of ≈100 mm^3^. After tumor volumes reached ≈100 mm^3^, mice were split into 3 groups (*n* = 5) to conduct the in vivo tumor reduction study. The mice in the 3 different groups were intravenously injected with 150 µL of PBS, 150 µL of ZW (50 nmol, 9.5 mg k^−1^g), or 150 µL of ZWPro (50 nmol, 9.5 mg k^−1^g) at 2 intervals (days 12 and 19). On every alternative day, the tumor size and total body weight of the mice were measured for up to 30 days. Thirty days after the treatment period, the mice were sacrificed, and tumor images were acquired.

### Histopathology Evaluation

To determine the tissue distribution of the NIR fluorophore kidney, liver, and tumor tissues were harvested 48 h after injection of ZW or ZWPro. Dissected tissues were embedded in a Tissue‐Tek optimum cutting temperature compound (OCT compound; Sakura Finetek, Torrance, CA, USA) without a prefixation step, and the tissue block was frozen at −80 °C. Frozen sections were cut at a thickness of 20 µm for the kidney and liver, and 5 µm for tumor tissue by a S Cryostat (Leica, No. CM3050). The tissue sections were then stained with hematoxylin and eosin. The Agilent BioTek Cytation. (Winooski, VT, USA) was used for pathological fluorescence imaging and observation.

### Statistical Analysis

GraphPad Prism 7 software was used to perform all statistical analyses as indicated in the figure captions. The fluorescence and background intensities of regions of interest study were quantified using the customized imaging software ImageJ. The TBR was calculated as TBR = Tumor fluorescence/background, where the background denotes the fluorescence intensity of muscle. All data depict the mean ± s.e.m. values with a minimum of 3 biological replicates. *p*‐values less than 0.05 were considered significant: *p < 0.05, **p < 0.01, ***p < 0.001, and ****p < 0.0001.

## Conflict of Interest

The authors declare no conflict of interest.

## Author Contributions

A.R. and J.S. contributed equally to this work. A.R. and J.S. performed the experiments. S.P. performed the chemical synthesis of fluorophores. A.R., J.S., and P.J. performed the animal study experiment. A.R., J.S., H.S.C., and H.C. reviewed, analyzed, and interpreted the data. A.R. and J.S. wrote the paper. All authors discussed the results and commented on the manuscript.

## Supporting information

Supporting Information

## Data Availability

The data that support the findings of this study are available from the corresponding author upon reasonable request.
