# Supplementary material for: Lysosome‐Targeted Bifunctional Therapeutics Induce Autodynamic Cancer Therapy
Source: Adv Sci (Weinh). 2024 Sep 4;11(41):2401424. doi: 10.1002/advs.202401424 (PMC11538690; doi:10.1002/advs.202401424)
Supplement: Supplementary file 1 — Supporting Information [file ADVS-11-2401424-s001.docx]

**Supporting information**

**Lysosome-targeted Bifunctional Therapeutics Induce Autodynamic Cancer Therapy**

Athira Raveendran^1*^, Jinhui Ser^1,2*^, Seung Hun Park^2^, Paul Jang^2^, Hak Soo Choi^2**^, Hoonsung Cho^1**^

^1^Department of Materials Science and Engineering, Chonnam National University, Gwangju 61186, Republic of Korea

^2^Gordon Center for Medical Imaging, Department of Radiology, Massachusetts General Hospital and Harvard Medical School, Boston, MA 02114, USA

* These authors contributed equally to this work

**Corresponding Authors: H.S.C. [hchoi12@mgh.harvard.edu](mailto:hchoi12@mgh.harvard.edu); or H.C. [cho.hoonsung@jnu.ac.kr](mailto:cho.hoonsung@jnu.ac.kr)

**Table of contents**

**Figure S1.**

**Figure S2.**

**Figure S3.**

**Figure S4.**

**Figure S5.**

**Figure S6.**

**Figure S7.**

**Figure S8.**

**
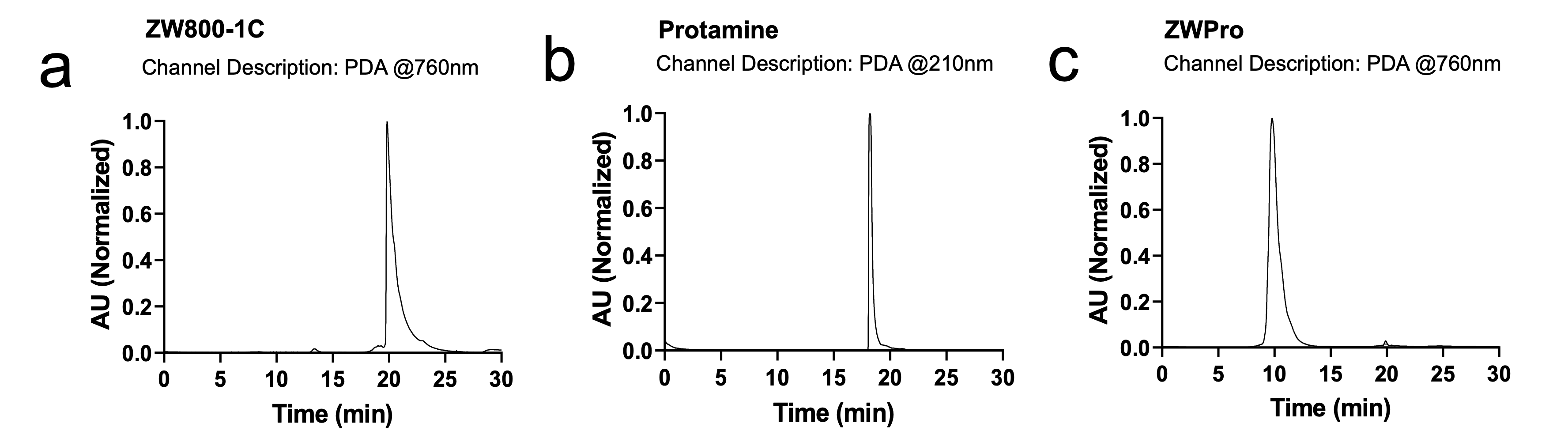
**

**Figure S1.** 1H NMR data of ZW800-1C, Protamine, and ZWPro.


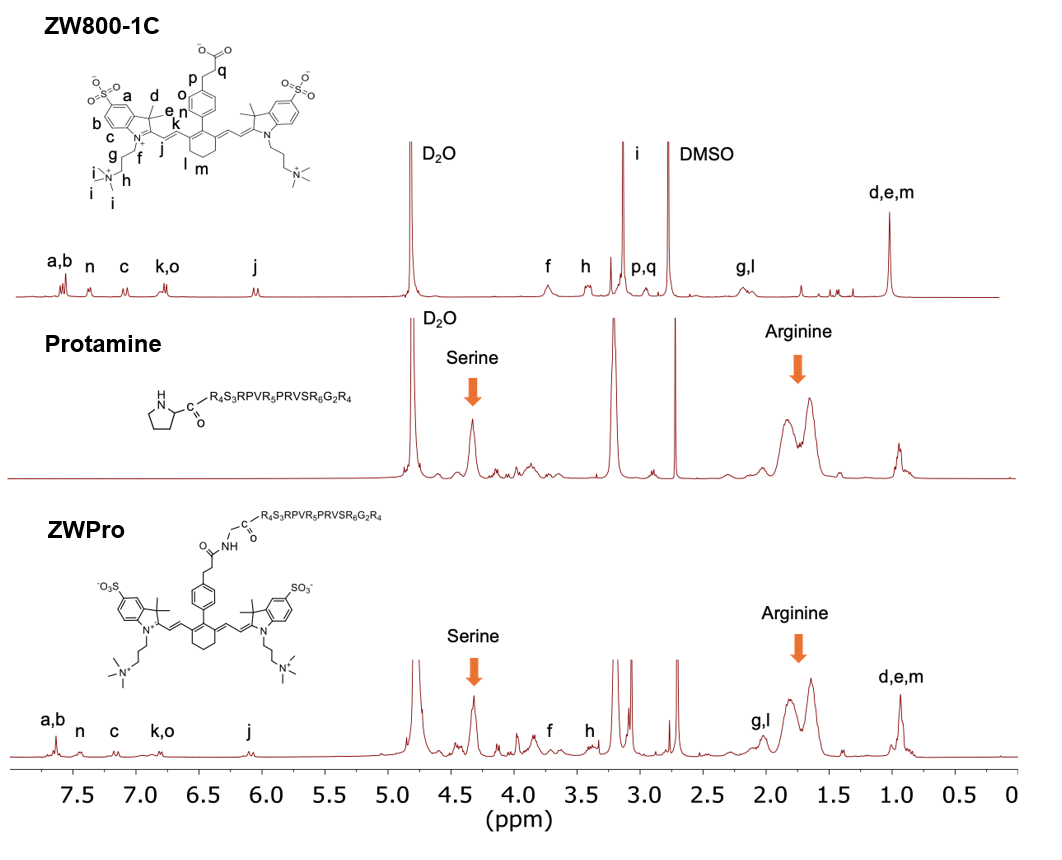


**Figure S2.** 1H NMR data of ZW800-1C, Protamine, and ZWPro.


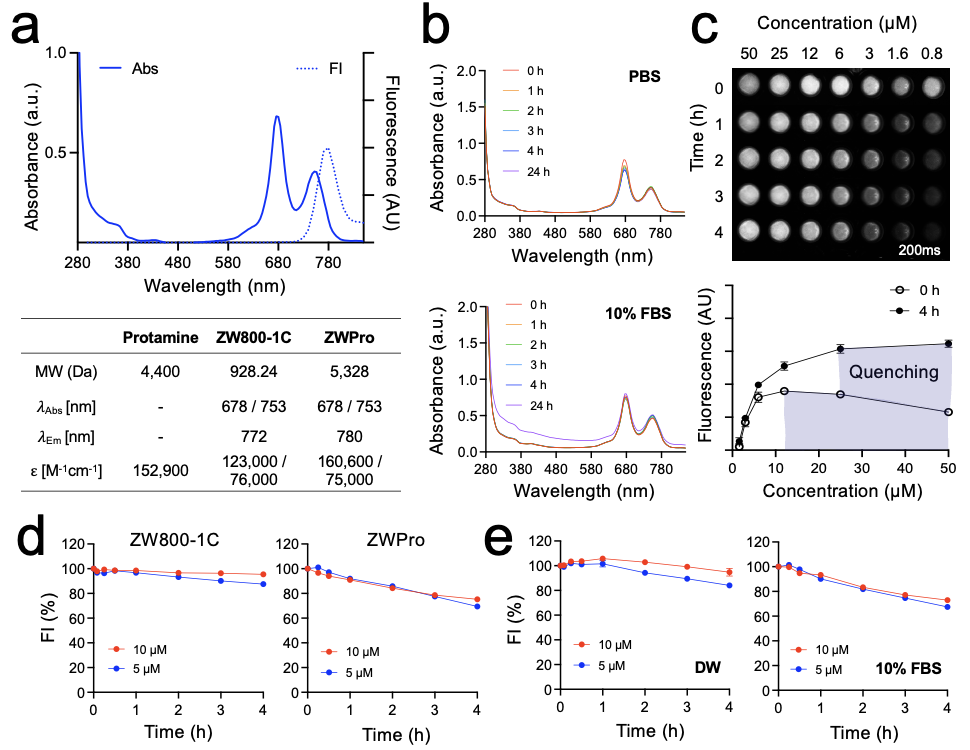


**Figure S3.** Optical property characterization of the ZWPro (a) Absorbance and Fluorescence spectra of ZWPro and optical properties of Pro, ZW800-1C, and ZWPro. (b) Stability test of ZWPro with PBS and 10% FBS in PBS until 24 h at 37^o^C incubator. (c) Fluorescence image and value are used to determine quenching concentration under a 760nm channel of different concentrations of ZWPro depending on time. Photostability test of (d) ZW800-1C and ZWPro in PBS. (e) Photostability test of ZWPro in different solvents. DW: Distilled water, 10% FBS: Fetal bovine serum in PBS. All photostability tests were conducted under the 760nm laser with 0.2mW^2^ power until 4h.


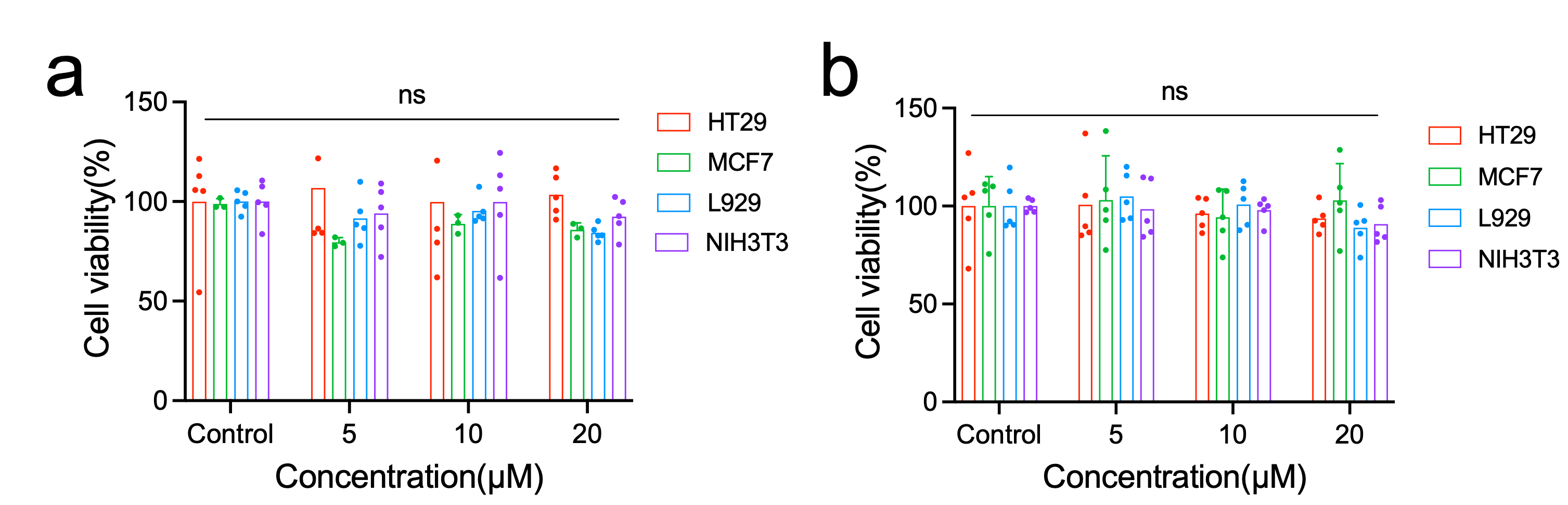


**Figure S4.** Cell cytotoxicity profiles of HT29, MCF7, L929, and NIH3T3 cells treated with 5, 10, and 20 μM of ZW (a) and protamine (b) for 24 h, followed by an assessment of cell viability using the MTT assay. Data are presented as mean ± standard deviation (n = 5) (ns, not significant by two-way analysis of variance followed by Turkey’s multiple comparisons test).


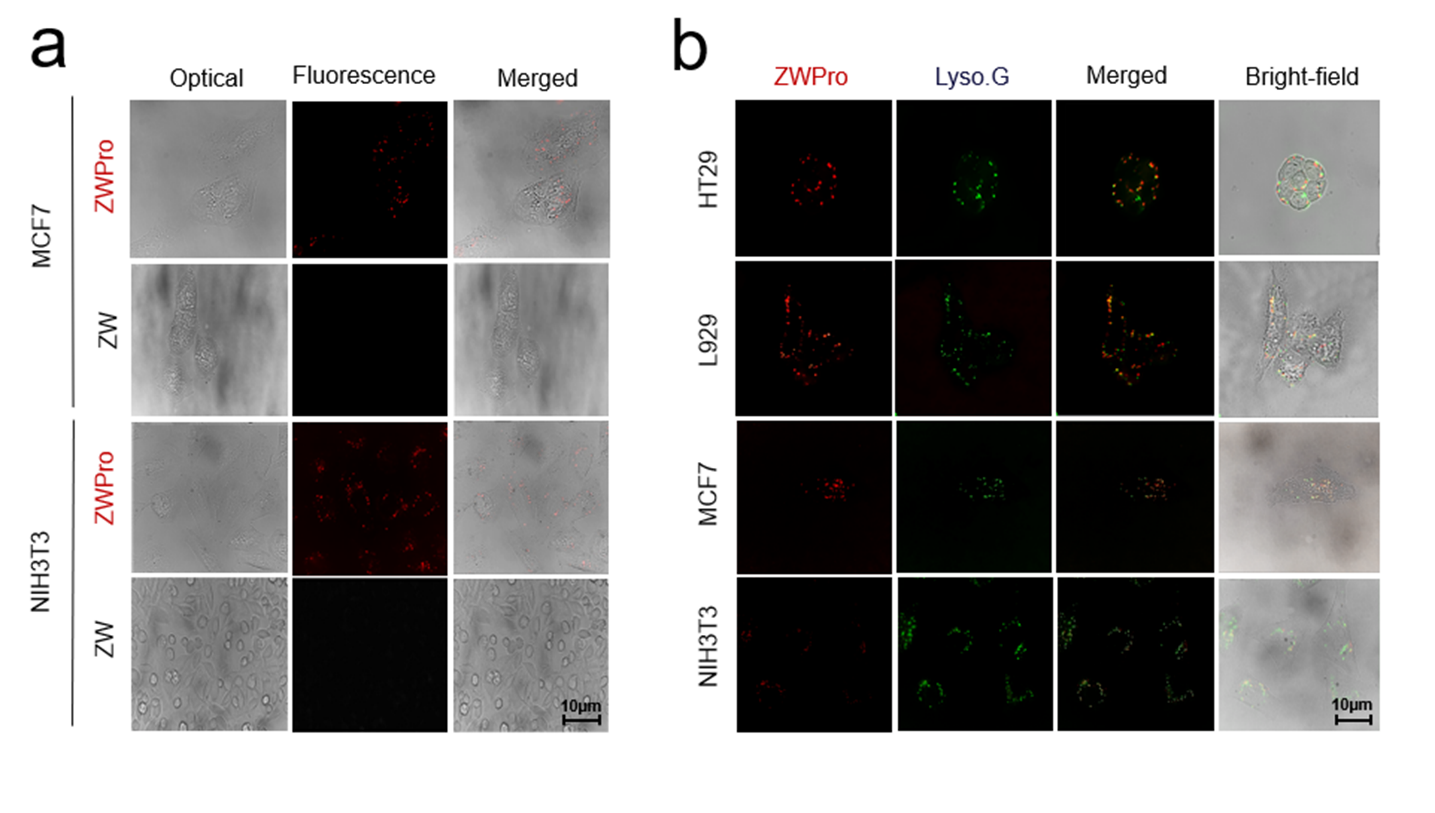


**Figure S5.** Cellular uptake and intracellular localization of ZW and ZWPro. (a) Uptake and intracellular endosomal localization of ZWPro and ZW (20 μM) in MCF7 and NIH3T3 cells were demonstrated using fluorescent microscopy. ZWPro appears to be internalized, while ZW does not show internalization. (b) Co-stained imaging outcomes using ZWPro (20 μM) and lysosomal marker (LysoTracker Green) in HT29, L929, MCF7, and NIH3T3 cells via fluorescent microscopy. Scale bar: 10 μm.


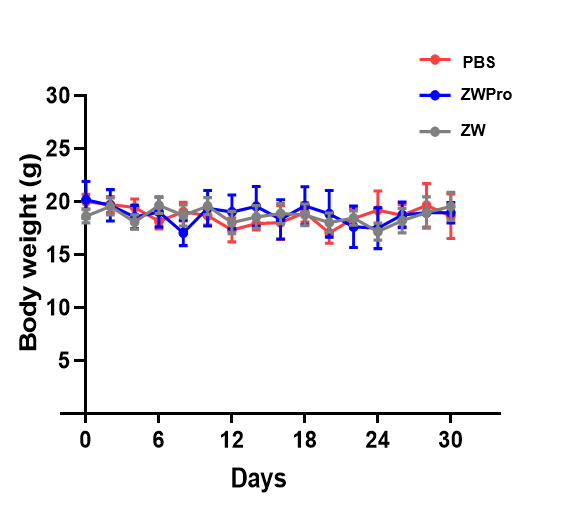


**Figure S6.** Body weight and tumor volume measurement changes in different groups.


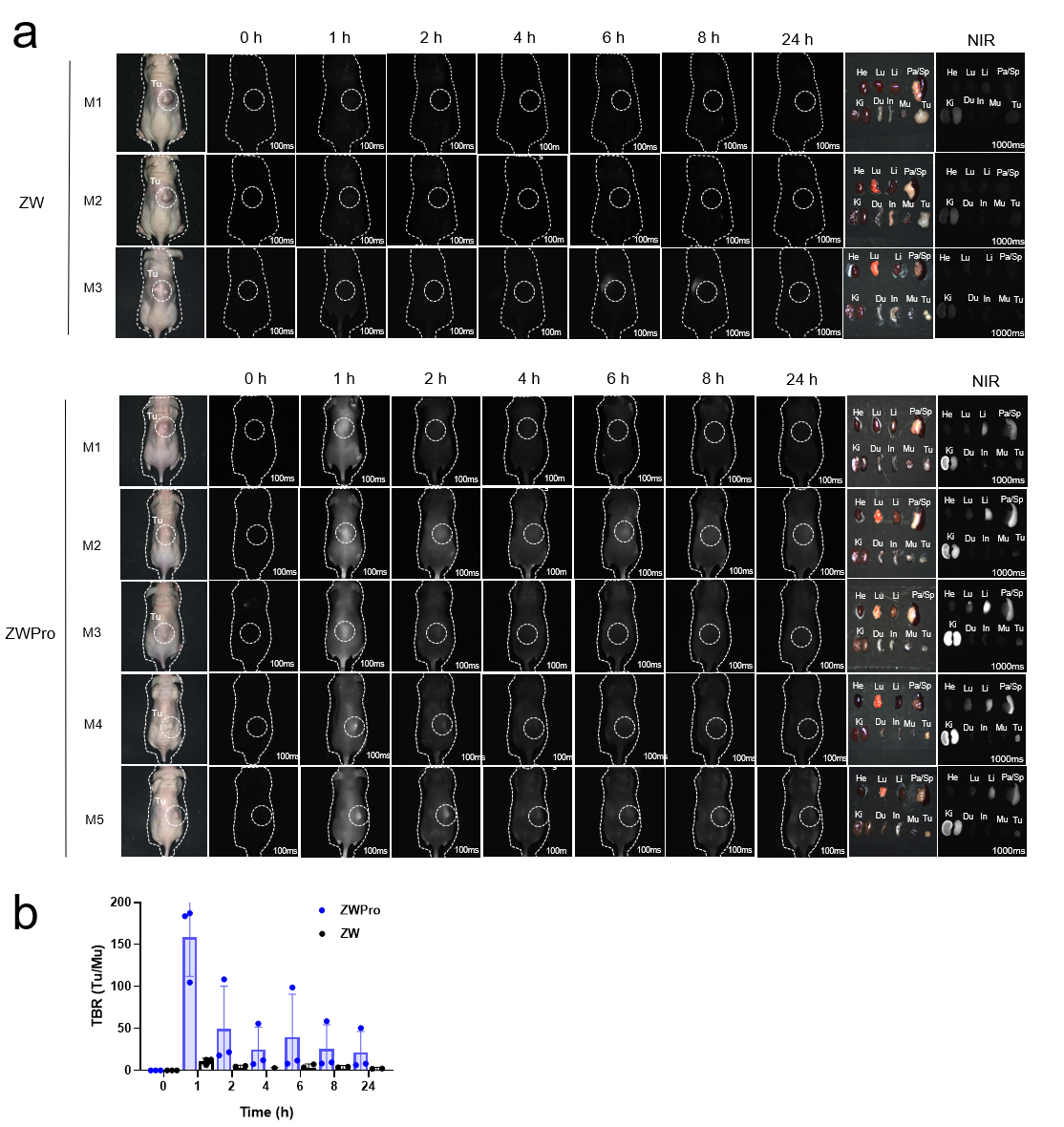


**Figure S7.** Near infrared (NIR) fluorescence images for each condition have identical exposure times. (a) ZW and ZWPro were injected (a total of 20 nmol) into BLAB/C nude mice (weights in the range of 20–25 g) and image time-dependent post-injection. Color and NIR fluorescence images of ZW and ZWPro (0.3 μmol kg^-1^) of dissected organs from treated mice at 48 h post-injection*.* NIR fluorescence images for each condition have identical exposure times. (Exposure time = 1000, 1500 ms, *n* = 3–5). Abbreviations: He, heart; Lu, lungs; Li, liver; Pa, pancreas; Sp, spleen; Ki, kidneys; In, intestine; Mu, muscle; Tu, tumor. Scale bar: 1 cm. (b) Tumor-to-background (TBR) signal ratios of the tumors compared with muscles for in vivo NIR imaging obtained from at each time point. Scale bar: 1 cm. Quantitative measurements of the fluorescence intensity of images (n = 3–5, means ± standard deviations). Exposure time = 100 ms, Scale bar =100 μm. (e) Body weight and tumor volume measurement changes in different groups (n=3).


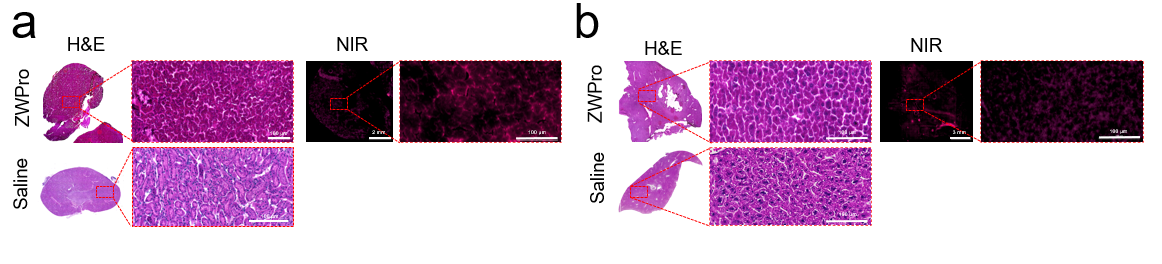


**Figure S8**. Bright field, NIR, and H&E staining images (20×) of kidney and liver from 20 nmol ZWPro and Saline injected mice.
